# Association of COVID-19 Case-Fatality Rate With State Health Disparity in the United States

**DOI:** 10.3389/fmed.2022.853059

**Published:** 2022-06-29

**Authors:** Yu-Che Lee, Ko-Yun Chang, Mehdi Mirsaeidi

**Affiliations:** ^1^Department of Medicine, University at Buffalo-Catholic Health System, Buffalo, NY, United States; ^2^Division of Chest Medicine, Taichung Veterans General Hospital, Taichung, Taiwan; ^3^Division of Pulmonary and Critical Care and Sleep Medicine, University of Florida College of Medicine, Jacksonville, FL, United States

**Keywords:** COVID-19, case-fatality rate, health disparity, COVID-19 vaccination, USA

## Abstract

**Background:**

The disproportionate burden of COVID-19 pandemic has become a major concern in the United States (US), but the association between COVID-19 case-fatality rate (CFR) and factors influencing health outcomes at a state level has not been evaluated.

**Methods:**

We calculated COVID-19 CFR for three different waves using COVID Data Tracker from the Centers for Disease Control and Prevention. America's Health Rankings assesses the factors that influence health outcomes to determine state's health rankings. The association between COVID-19 CFR and state health disparities was analyzed by linear regression.

**Results:**

States with better rankings of Physical Environment were associated with lower CFR for the 1st wave (β = 0.06%, R^2^ = 0.170, *P* = 0.003). There was a paradoxical association between the 2nd wave CFR and Clinical Care (β = −0.04%, R^2^ = 0.112, *P* = 0.017) and Overall health rankings (β = −0.03%, R^2^ = 0.096, *P* = 0.029). For the 3rd wave, states with better rankings of Overall health factors (β = 0.01%, R^2^ = 0.179, *P* = 0.002), Social & Economic Factors (β = 0.01%, R^2^ = 0.176, *P* = 0.002), Behaviors (β = 0.01%, R^2^ = 0.204, *P* < 0.001), and Health Outcomes (β = 0.01%, R^2^ = 0.163, *P* = 0.004) were associated with lower CFR. COVID-19 vaccination coverage was also associated with state health rankings (at least one dose: β = −0.13%, R^2^ = 0.305, *P* < 0.001; fully vaccinated: β = −0.06%, R^2^ = 0.120, *P* = 0.014).

**Conclusions:**

These findings suggested targeted public health interventions and mitigation strategies addressing health disparities are essential to improve inequitable outcomes of COVID-19 in the US.

## Introduction

The novel Coronavirus Disease 2019 (COVID-19), which is caused by the severe acute respiratory syndrome coronavirus 2 (SARS-CoV-2) and first discovered in Wuhan, China, on December 31, 2019, has continued to spread in the United States (US) and overwhelm healthcare systems all over the world (1–4). As of May 1, 2022, there are more than 510 million confirmed cases and 6 million deaths from COVID-19 worldwide (5). In the US, the impact of racial/ethnic and socioeconomic disparities on COVID-19 incidence, mortality and outcomes has been well established, with racial/ethnic minority groups consistently having higher incidence, mortality rates and adverse outcomes driven by sociodemographic disadvantage (6–10). The disproportionate and inequitable burden of the COVID-19 pandemic has become a major concern and emerging evidence on COVID-19 disparities is vitally important for guiding public health policy and healthcare resource allocation in the US (11).

Case-fatality rate (CFR), defined as the proportion of deaths among incident cases of a disease, has been used to investigate and compare the severity and outcomes of COVID-19, as well as the efficacy of treatment and healthcare capacity in response to the pandemic between countries (12–15). Prior literature also indicated that COVID-19 CFR was significantly higher in minority groups and rural counties and associated with socioeconomic status in the US (16, 17).

America's Health Rankings (AHR), a partnership of the United Health Foundation and the American Public Health Association, is an annual comprehensive assessment of the nation's health on a state-by-state basis since 1990. AHR assesses the factors that influence health outcomes and uses different measures to determine the state's health rankings, with “1” being the healthiest state and “50” the most unhealthy state.

Despite several studies focusing on the variation of impact on COVID-19 pandemic across the US, little is known about the role of the state-level health disparity in COVID-19 outcomes. In this study, we hypothesized that COVID-19 CFR correlated with state health rankings and COVID-19 vaccination coverage, which reflect the healthcare and socioeconomic disparities between states. We aimed to examine the association of COVID-19 CFR, state health rankings, and COVID-19 vaccination rates to provide another perspective of the impact between COVID-19 and health disparities in the US.

## Materials and Methods

### Data Resources

COVID-19 confirmed cases and deaths from March 25, 2020 to April 1, 2021 were obtained from the COVID Data Tracker provided by the Centers for Disease Control and Prevention (CDC). CFR calculations were performed by dividing the number of confirmed deaths by the confirmed cases at the same time-period expressed as a percentage, where values approaching 0% represent an excellent disease outcome (lower severity) and those approaching 100% represent a poor outcome (higher severity). With these data, we calculated three specific CFR in different time-period for all 50 states: March 25 to June 3, 2020 as the “1st wave”, June 4 to September 2, 2020 as the “2nd wave”, and September 3, 2020 to April 1, 2021 as the “3rd wave” of COVID-19.

The state health rankings were calculated by a formula using different weighted measures in five main categories: 20% for Behaviors (Insufficient Sleep, Nutrition and Physical Activity, Sexual Health, Tobacco Use), 30% for Social and Economic Factors (Community and Family Safety, Economic Resources, Education, Social Support and Engagement), 10% for Physical Environment (Air and Water Quality, Housing and Transit), 15% for Clinical Care (Assess to Care, Preventive Clinical Services, Quality of Care), and 25% for Health Outcomes (Behavioral Health, Mortality, Physical Health). The Overall health rankings in 2020 and each category of rankings for Behaviors, Social and Economic Factors, Physical Environment, Clinical Care, and Health Outcomes were examined in the current study as we previously described (18, 19).

In addition, the percentages of people who received at least one dose of COVID-19 vaccine and fully vaccinated by state until April 1, 2021 were obtained from the CDC.

### Statistical Analyses

Scatterplots of Overall health rankings, five core measures of health factor rankings by state, and the percentages of COVID-19 vaccinations vs. CFR were generated, respectively. Linear regression analyses were performed with the following formulas to calculate the association between state health rankings, COVID-19 vaccinations, and CFR: CFR = Overall Health Rankings ^*^ β + α; CFR = Behaviors rankings ^*^ β + α; CFR = Social & Economic Factors rankings ^*^ β + α; CFR = Physical Environment rankings ^*^ β + α; CFR = Clinical Care rankings ^*^ β + α; CFR = Health Outcomes rankings ^*^ β + α; CFR = COVID-19 vaccinations ^*^ β + α. Separate regression models were used to avoid potential collinearity issues. Data management and statistical analyses were performed using Microsoft Excel and Statistics Software SAS. ^®^ *P*-values < 0.05 using two-sided *T*-tests indicated statistical significance.

## Results

### COVID-19 Case Fatality Rate by State

**Figure 1:**
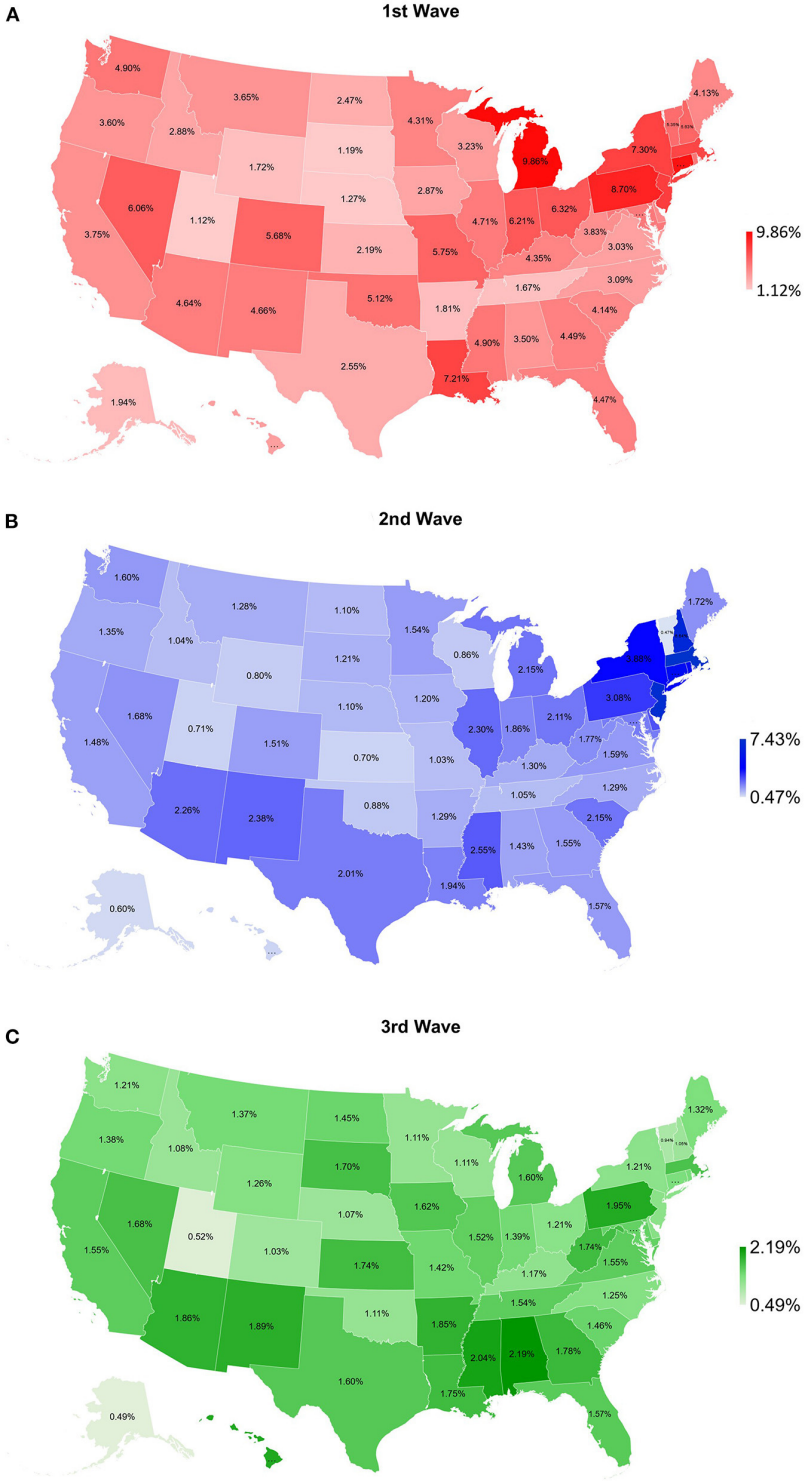
Map of COVID-19 case-fatality rate for **(A)** 1st wave (March 25–June 3, 2020), **(B)** 2nd wave (June 4–September 2, 2020), and **(C)** 3rd wave (September 3, 2020–April 1, 2021) for 50 states of the US.

From the beginning of COVID-19 pandemic to April 1, 2021, a total of 30,481,267 people were diagnosed with COVID-19 and 558,761 people died from COVID-19 in the US. The incidence, mortality, and CFR were 9,172 per 100,000 population, 168 per 100,000, and 1.8%, respectively. The confirmed cases, deaths, and CFRs in three different time-period were 1,923,996, 110,479, and 5.7% (1st wave), 4,335,732, 81,265, and 1.9% (2nd wave), and 24,221,539, 367,017, and 1.5% (3rd wave). There was a remarkable geographic difference in CFRs by state. During the 1st wave, Michigan had the highest CFR (9.9%) whereas Utah had the lowest CFR (1.1%). The highest CFR for the 2nd wave was recorded in Massachusetts (7.4%) and the lowest in Vermont (0.5%). Regarding the 3rd wave, the lowest CFR was reported in Alaska (0.5%) and the highest in Alabama (2.2%). The results are summarized in Table 1 and shown in Figure 1.

**Table 1:** Summary of COVID-19 case-fatality rate, state health rankings, and COVID vaccination rate of 50 states, as of April 1, 2021.

**State**	**COVID-19 case-fatality rate (%)**			**2020 America's health rankings (AHR)**						**COVID-19** **vaccination rate (%)**	
	**1st wave** **(March 25–June 3, 2020)**	**2nd wave** **(Jun 4–September 2, 2020)**	**3rd wave** **(September 3, 2020–April 1, 2021)**	**Overall**	**Behaviors**	**Social and economic factors**	**Physical environment**	**Clinical care**	**Health outcomes**	**At least one dose**	**Fully vaccinated**
Alabama	3.50	1.43	2.19	46	46	43	23	43	48	24.2	13.7
Alaska	1.94	0.60	0.49	29	32	36	40	34	11	34.2	22.6
Arizona	4.64	2.26	1.86	32	24	35	26	39	29	30.1	17.4
Arkansas	1.81	1.29	1.85	48	45	48	11	42	47	26.6	14.3
California	3.75	1.48	1.55	20	14	27	49	23	5	31.3	16.4
Colorado	5.68	1.51	1.03	9	7	13	5	17	9	30.5	17.9
Connecticut	9.36	4.77	1.32	4	3	15	33	5	3	36.0	20.9
Delaware	4.00	2.69	1.23	23	30	16	18	16	35	31.8	16.8
Florida	4.47	1.57	1.57	33	31	29	36	41	27	28.5	16.3
Georgia	4.49	1.55	1.78	38	39	30	10	47	37	24.3	12.5
Hawaii	3.12	0.68	1.96	7	13	11	28	4	1	32.2	19.5
Idaho	2.88	1.04	1.08	22	17	23	30	37	14	26.3	16.8
Illinois	4.71	2.30	1.52	26	23	24	32	24	28	31.8	16.9
Indiana	6.21	1.86	1.39	36	36	32	27	38	36	25.8	17.0
Iowa	2.87	1.20	1.62	11	22	5	15	9	15	31.3	19.7
Kansas	2.19	0.70	1.74	24	26	21	35	28	26	30.5	17.2
Kentucky	4.35	1.30	1.17	44	48	34	19	31	46	31.5	17.9
Louisiana	7.21	1.94	1.75	50	50	50	48	40	50	26.6	16.8
Maine	4.13	1.72	1.32	14	20	12	13	7	23	35.2	20.2
Maryland	4.80	2.05	1.49	10	14	18	8	10	8	31.8	17.8
Massachusetts	7.10	7.43	1.67	2	9	7	29	1	2	34.9	19.5
Michigan	9.86	2.15	1.60	34	35	44	41	15	40	29.2	17.4
Minnesota	4.31	1.54	1.11	5	4	6	4	12	7	31.9	19.3
Mississippi	4.90	2.55	2.04	49	49	47	47	49	49	24.7	15.2
Missouri	5.75	1.03	1.42	39	43	26	25	35	38	25.9	15.6
Montana	3.65	1.28	1.37	28	8	25	22	32	41	31.3	19.7
Nebraska	1.27	1.10	1.07	17	18	17	21	13	20	31.6	19.3
Nevada	6.06	1.68	1.68	37	38	31	14	48	30	28.3	16.3
New Hampshire	5.63	6.64	1.05	1	5	1	24	6	17	36.8	18.2
New Jersey	7.45	7.08	1.21	13	11	3	50	26	4	34.1	19.5
New Mexico	4.66	2.38	1.89	42	34	49	31	29	31	38.7	24.0
New York	7.30	3.88	1.21	21	19	37	34	19	10	31.4	17.3
North Carolina	3.09	1.29	1.25	30	36	22	9	27	31	29.3	16.9
North Dakota	2.47	1.10	1.45	16	28	10	1	18	16	32.8	20.6
Ohio	6.32	2.11	1.21	41	41	42	39	29	39	29.6	17.2
Oklahoma	5.12	0.88	1.11	47	46	45	45	46	43	31.4	18.5
Oregon	3.60	1.35	1.38	19	12	20	42	22	19	28.7	17.1
Pennsylvania	8.70	3.08	1.95	27	33	27	46	8	34	32.4	17.2
Rhode Island	4.92	4.49	1.36	12	16	18	38	2	18	33.2	21.5
South Carolina	4.14	2.15	1.46	40	40	41	7	36	42	27.7	15.4
South Dakota	1.19	1.21	1.70	25	27	39	6	21	24	35.4	22.9
Tennessee	1.67	1.05	1.54	43	42	40	17	44	44	25.6	14.2
Texas	2.55	2.01	1.60	35	29	33	43	50	22	26.4	14.3
Utah	1.12	0.71	0.52	6	2	2	12	25	6	26.5	11.9
Vermont	5.35	0.47	0.94	3	1	9	20	3	12	34.0	19.2
Virginia	3.03	1.59	1.55	15	21	8	2	20	21	31.9	17.5
Washington	4.90	1.60	1.21	8	6	4	16	14	13	30.3	18.4
West Virginia	3.83	1.77	1.74	45	44	46	44	33	45	30.6	19.4
Wisconsin	3.23	0.86	1.11	18	10	14	37	11	33	32.5	19.0
Wyoming	1.72	0.80	1.26	31	25	38	3	45	25	27.8	18.8

### Association Between State Health Rankings and COVID-19 CFR

**Figure 2:**
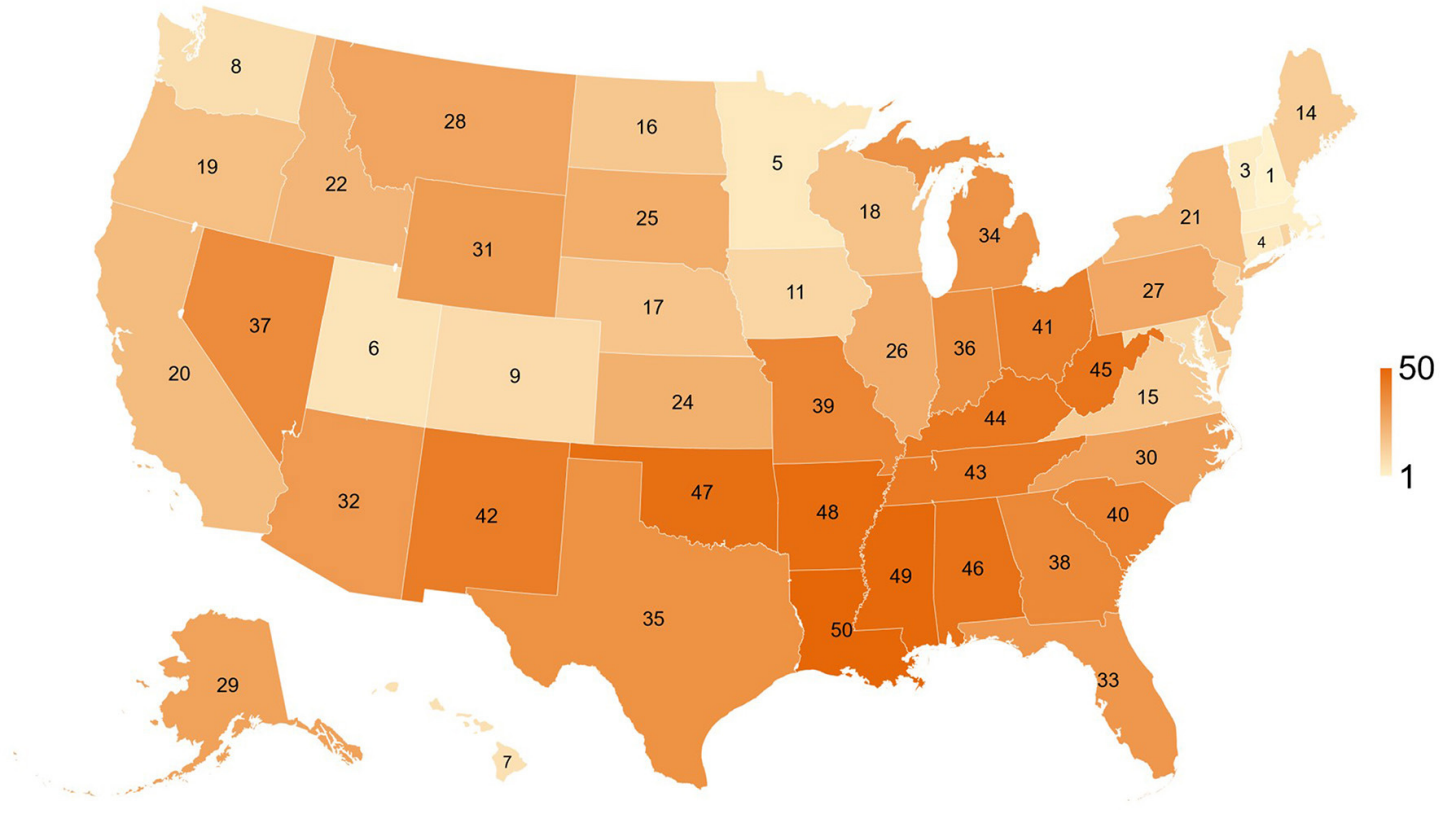
Map of state overall health rankings for 50 states of the US, 2020.

**Figure 3:**
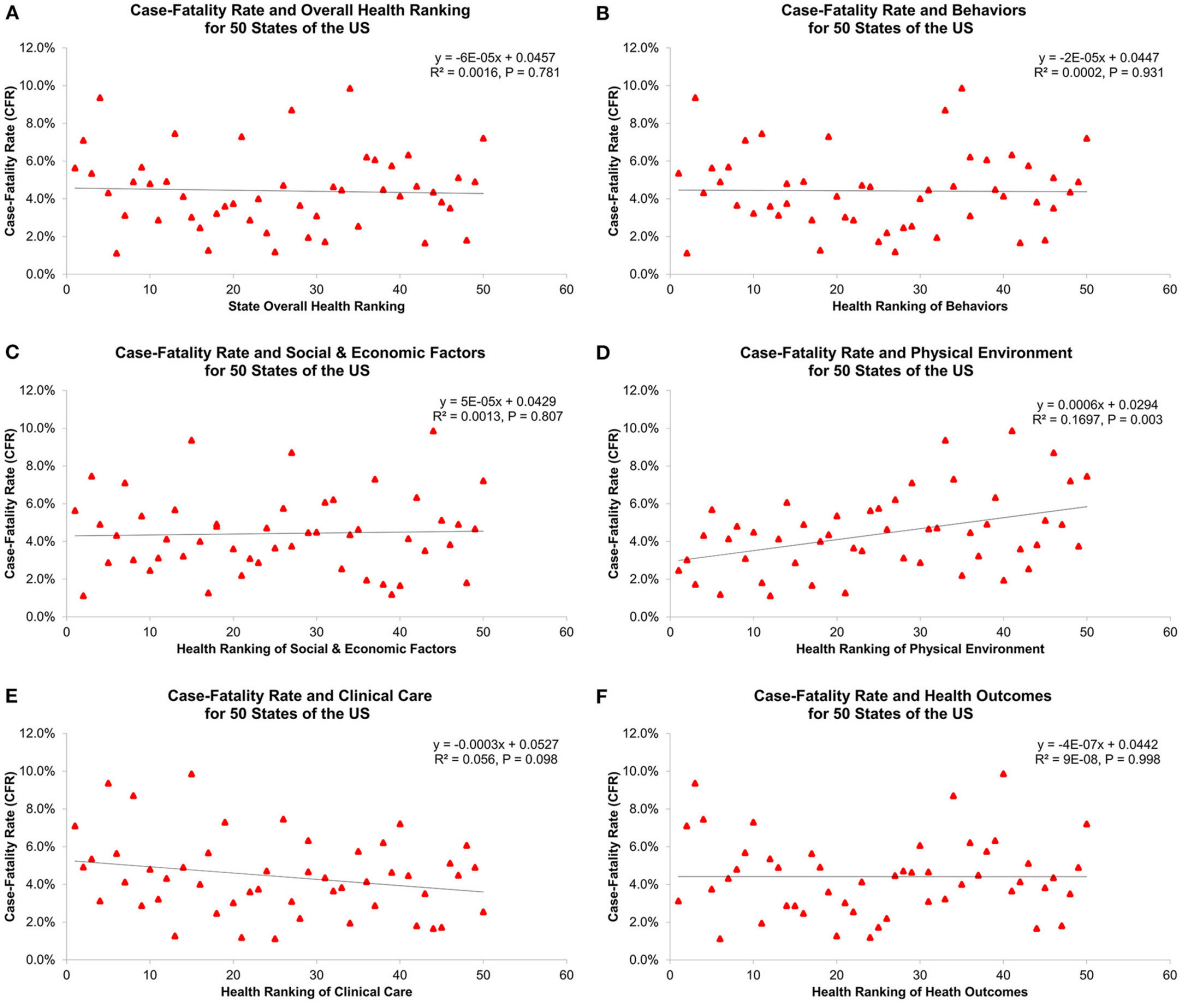
Association of 1st wave COVID-19 case-fatality rate with **(A)** overall health ranking, **(B)** behaviors, **(C)** social and economic factors, **(D)** physical environment, **(E)** clinical care, and **(F)** health outcomes for 50 states of the US.

**Figure 4:**
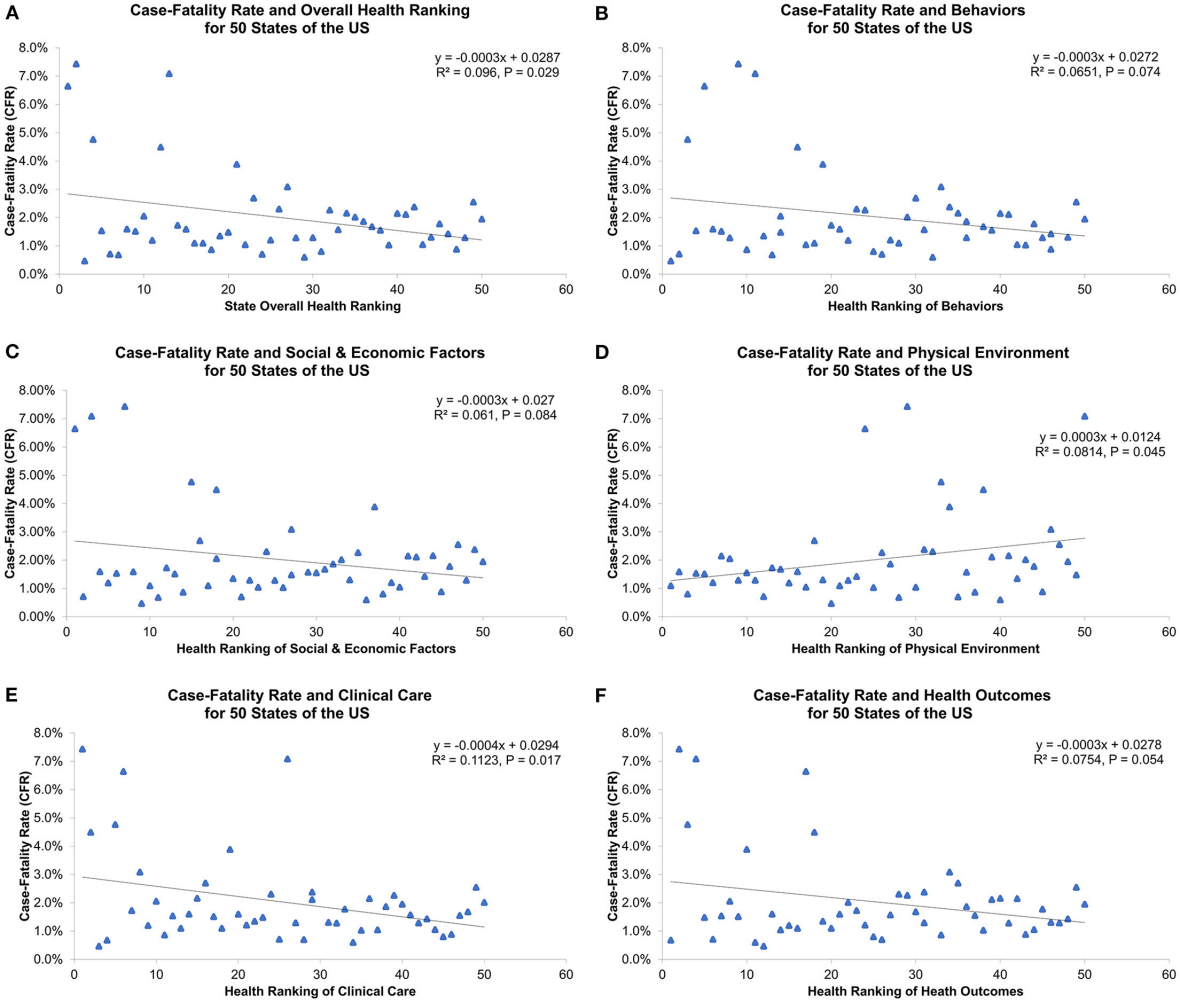
Association of 2nd wave COVID-19 case-fatality rate with **(A)** overall health ranking, **(B)** behaviors, **(C)** social and economic factors, **(D)** physical environment, **(E)** clinical care, and **(F)** health outcomes for 50 states of the US.

**Figure 5:**
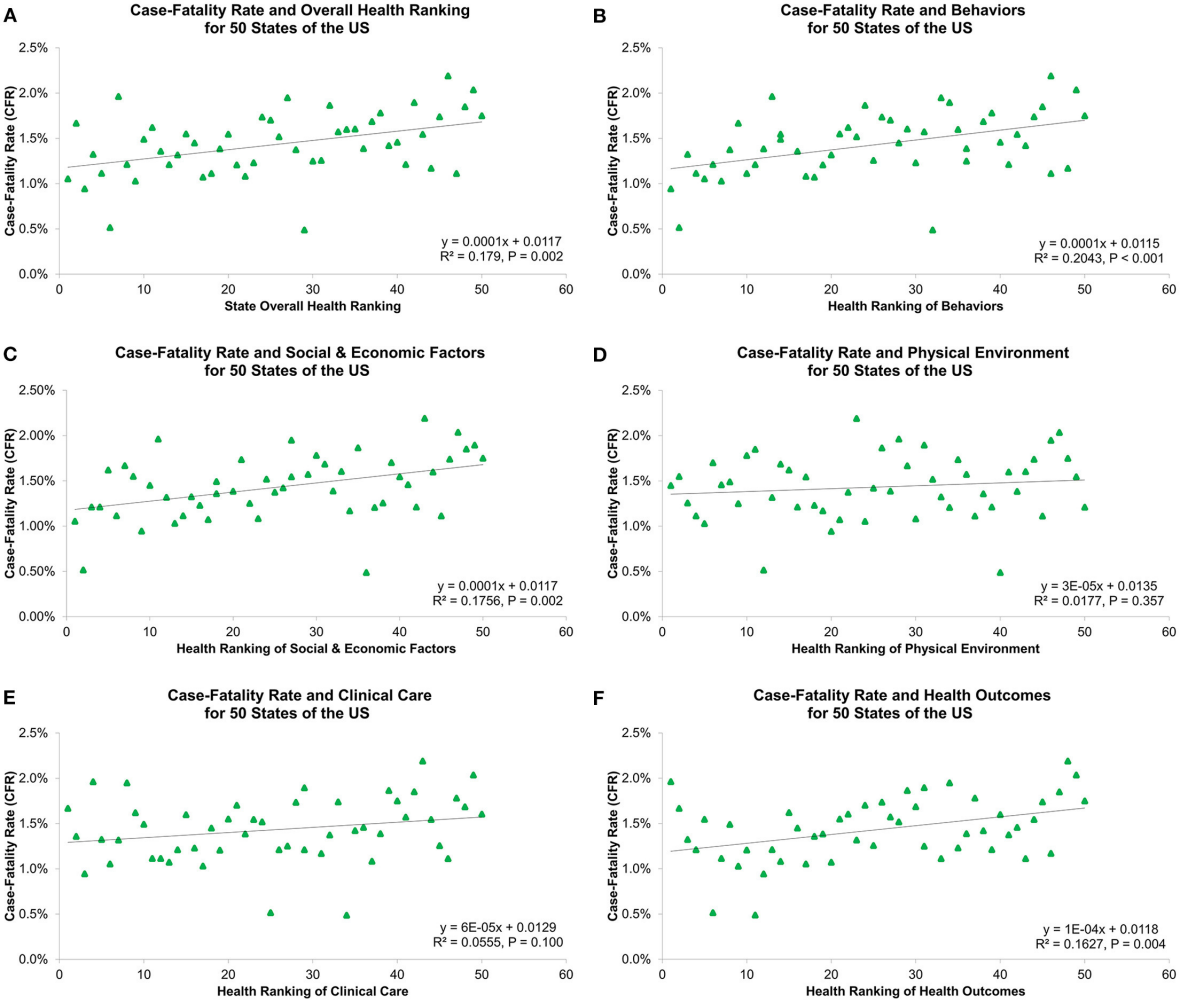
Association of 3rd wave COVID-19 case-fatality rate with **(A)** overall health ranking, **(B)** behaviors, **(C)** social and economic factors, **(D)** physical environment, **(E)** clinical care, and **(F)** health outcomes for 50 states of the US.

Table 1 and Figure 2 show overall health rankings and five main health factors rankings in 2020 for 50 states. Among 50 states, New Hampshire had the highest ranking for Overall health factors and Social and Economic Factors, whereas Louisiana had the lowest ranking for Overall health factors, Social and Economic Factors, Behaviors, and Health Outcomes. During the 1st wave, states with better health rankings of Physical Environment were associated with lower CFR (β = 0.06%, R^2^ = 0.170, *P* = 0.003, Figure 3). For the 2nd wave, states with better health rankings of Physical Environment were also associated with lower CFR (β = 0.03%, R^2^ = 0.081, *P* = 0.045), whereas an opposite association was found in Clinical Care (β = −0.04%, R^2^ = 0.112, *P* = 0.017) and Overall health rankings (β = −0.03%, R^2^ = 0.096, *P* = 0.029, Figure 4). Regarding the 3rd wave, states with better health rankings of Overall health factors (β = 0.01%, R^2^ = 0.179, *P* = 0.002, Figure 5), Social and Economic Factors (β = 0.01%, R^2^ = 0.176, *P* = 0.002), Behaviors (β = 0.01%, R^2^ = 0.204, *P* < 0.001), and Health Outcomes (β = 0.01%, R^2^ = 0.163, *P* = 0.004) were all associated with lower CFR. This is the most important finding of our study result which showed state health rankings of different health indicators were significantly associated with COVID-19 CFR during these three different waves of pandemic.

### Association Between COVID-19 Vaccinations and COVID-19 CFR

**Figure 6:**
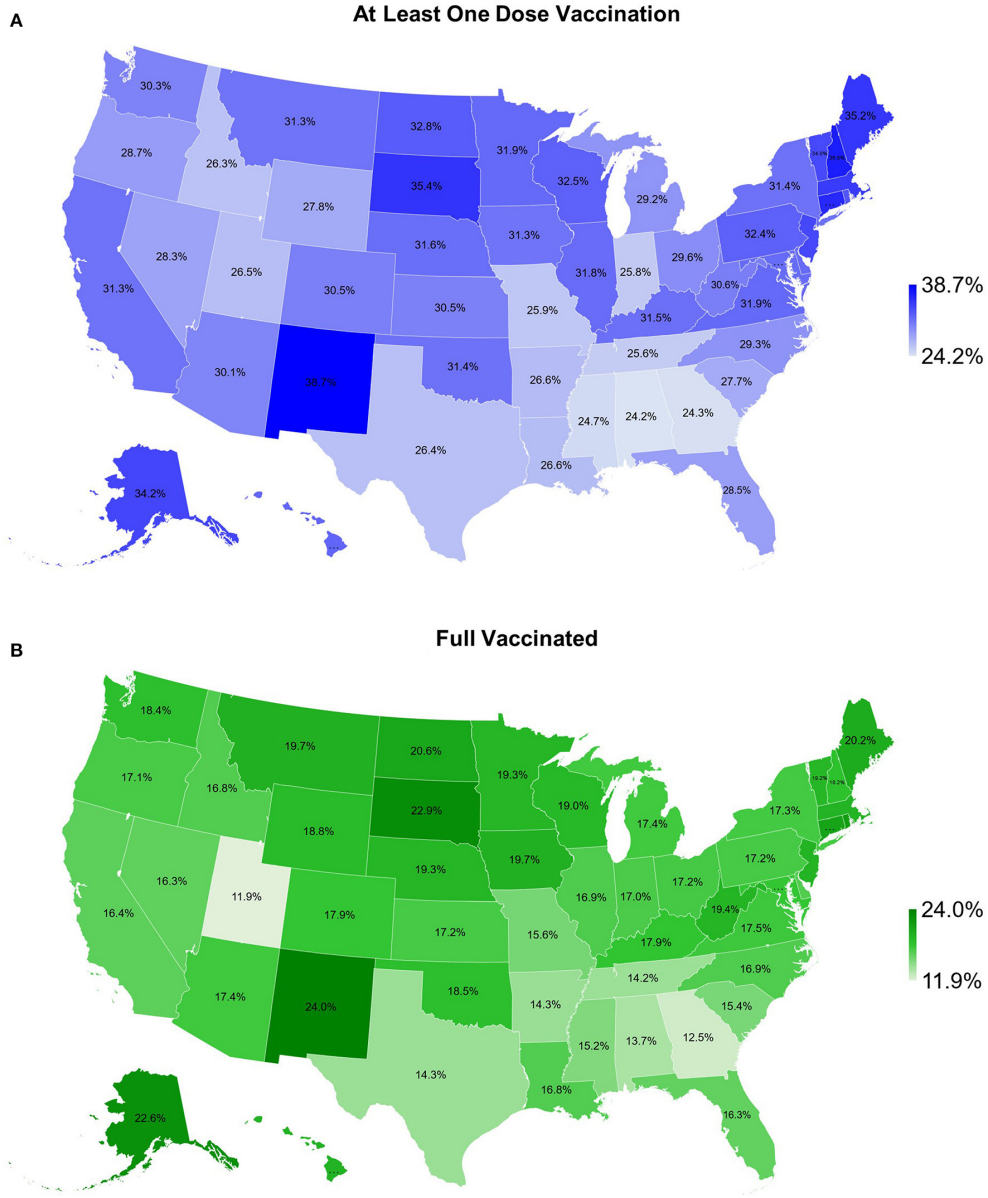
Map of COVID-19 vaccination rate for **(A)** as least one dose vaccination and **(B)** fully vaccinated for 50 states of the US, as of April 1, 2021.

**Figure 7:**
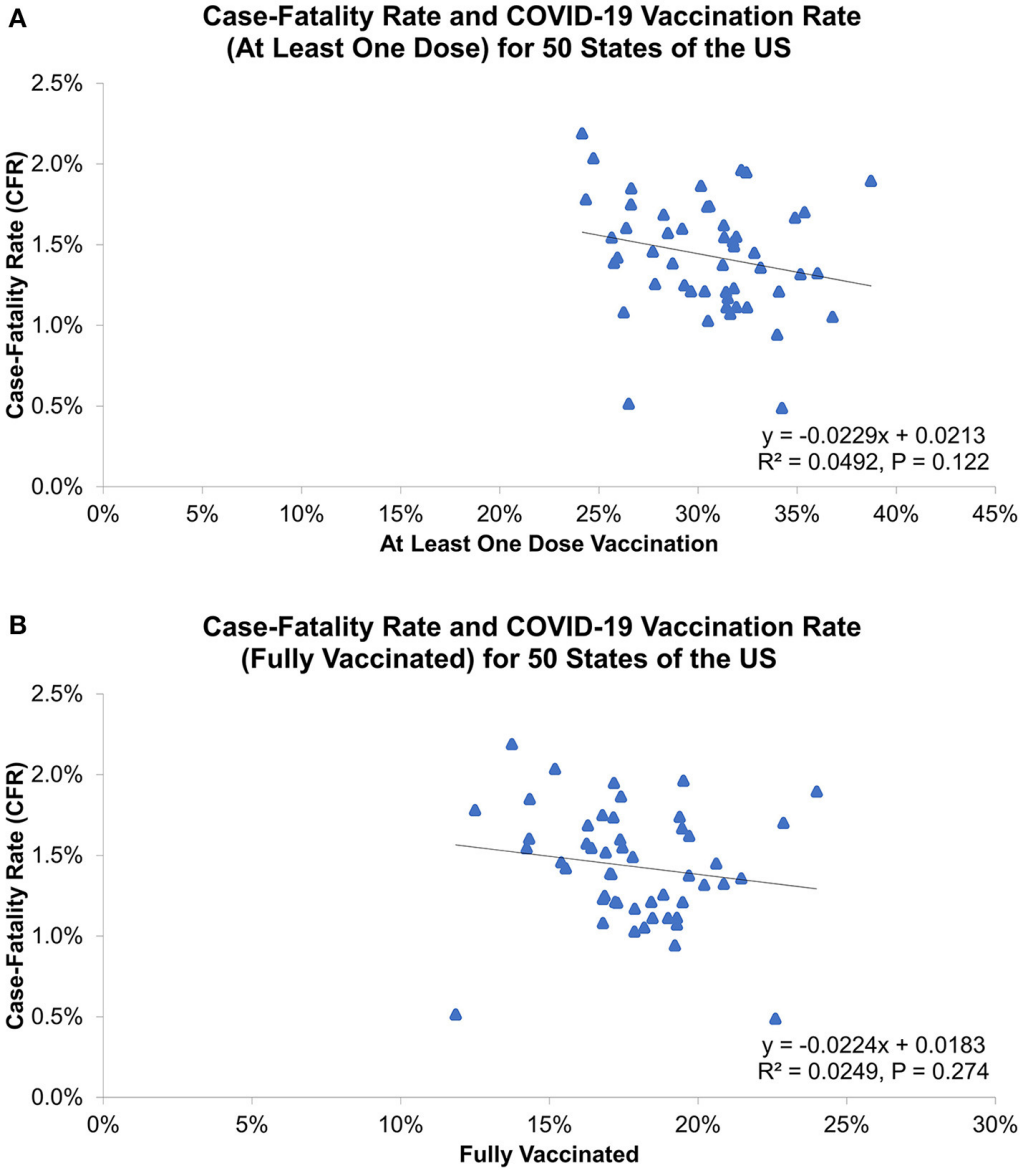
Association of 3rd wave COVID-19 case-fatality rate with vaccination rate for **(A)** as least one dose vaccination and **(B)** fully vaccinated, as of April 1, 2021.

Table 1 and Figure 6 show COVID-19 vaccination rates for 50 states. The highest rates for at least one dose of vaccination and fully vaccinated were both in New Mexico (38.7 and 24.0%), but the lowest rates for at least one vaccination and fully vaccinated were in Alabama (24.2%) and Utah (11.9%), respectively. There was no association between COVID-19 vaccinations and the 3rd wave CFR (at least one dose: R^2^ = 0.049, *P* = 0.122; fully vaccinated: R^2^ = 0.025, *P* = 0.274, Figure 7).

## Discussion

To our knowledge, this is the first study to investigate the association between COVID-19 CFR and state health factors in the US. This study clearly shows geographic differences in COVID-19 outcomes between states and its significant association with state-level health disparities. During different pandemic waves, state-level COVID-19 CFRs were significantly associated with different health indicators, including Overall health factors, Social and Economic Factors, Behaviors, and Health Outcomes. The mapping of COVID-19 CFR demonstrates distinct distributions for the three waves. The highest CFRs for the 1st and 2nd waves were found in some Northeastern states (New York, New Jersey, Massachusetts, Connecticut, and Pennsylvania), Michigan and Louisiana, whereas the lowest in Utah, South Dakota and Nebraska. For the 3rd wave, the highest CFRs were seen in some Southern states (Alabama, Mississippi, Arkansas, Georgia, and Louisiana), Hawaii and Pennsylvania, but the lowest in Alaska, Utah and Vermont. These geographic findings align with the official COVID-19 reports from CDC and Johns Hopkins University Center for Systems Science and Engineering (20), indicating COVID-19 confirmed cases and deaths in the early months of the pandemic were mostly concentrated in the metropolitan areas of New York (New York City), Louisiana (New Orleans), Massachusetts (Boston) and Michigan (Detroit), and the geography has shifted as COVID-19 spread across the US over time (21).

Geographic difference in COVID-19 cases, deaths and CFRs likely reflects variations in epidemiologic factors, clinical practices, and public health interventions. During the 1st and 2nd wave, we found a positive association between health rankings of Physical Environment and COVID-19 CFRs, suggesting unfavorable COVID-19 outcomes in states with worse physical environments. Since COVID-19 is primarily transmitted by exposure to infectious respiratory droplets and there were no effective treatments identified during these two waves, population density and mitigation strategies to reduce transmission might contribute to observed variation in COVID-19 infection rates and disease outcomes (21, 22). In our study, air quality and severe housing problems with overcrowding occupants and inadequate ventilation are the main measures of Physical Environment indicator. These factors can be the possible explanation for our study result as greater household crowding and poor indoor ventilation with outside air have shown to increase indoor airborne contaminants, the risk of COVID-19 infection and associated outcomes (16, 23, 24). In contrast, a surprising and interesting finding was the paradoxical relationship between COVID-19 CFRs and Clinical Care indicator and Overall health rankings during the 2nd wave. The negative association in this wave could be attributed to differences in COVID-19 testing availabilities and capacities across the US (25). This suggested that states with better Clinical Care and Overall health rankings may have greater access and abilities to test and identify cases, leading to higher testing volume, more precise calculation for incidence and mortality, and different outcomes of COVID-19.

During the 3rd wave, better understanding of the disease, more clear treatment guidelines, expanded medical resources and workforce, higher implementation of mitigation strategies, and vaccine development and administration have resulted in reduced disease severity and improved outcomes of COVID-19 (26). In this wave, our study demonstrated a positive association between state health rankings of Overall health factors, Social & Economic Factors, Behaviors, Health Outcomes and COVID-19 CFRs. Previous studies have identified that racial/ethnic health disparities and socioeconomic status, including poverty, unemployment, income inequality, education, occupation and uninsured rate have significant impacts on incidence, mortality and clinical outcomes of COVID-19 (27–34). Specifically, Dasgupta et al., Nayak et al., Karaye et al., Khazanchi et al., and Karmakar et al. all found that higher Social Vulnerability Index (SVI, a validated measure using four domains: Socioeconomic Status, Household Composition and Disability, Racial/Ethnic Minority and Language, and Housing and Transportation) was associated with greater COVID-19 incidence, mortality and adverse outcomes (16, 24, 35–37). Interestingly, some studies also indicated that limited English proficiency and socioeconomic disadvantage in social distancing are the important factors resulting in wide-ranging differences in the impact of COVID-19 pandemic across the US (24, 38). Taken together, all these findings are generally consistent with our study result as states with better overall health and socioeconomic status may have superior health care system, public health infrastructures and policies, contributing to favorable COVID-19 outcomes in the US.

**Figure 8:**
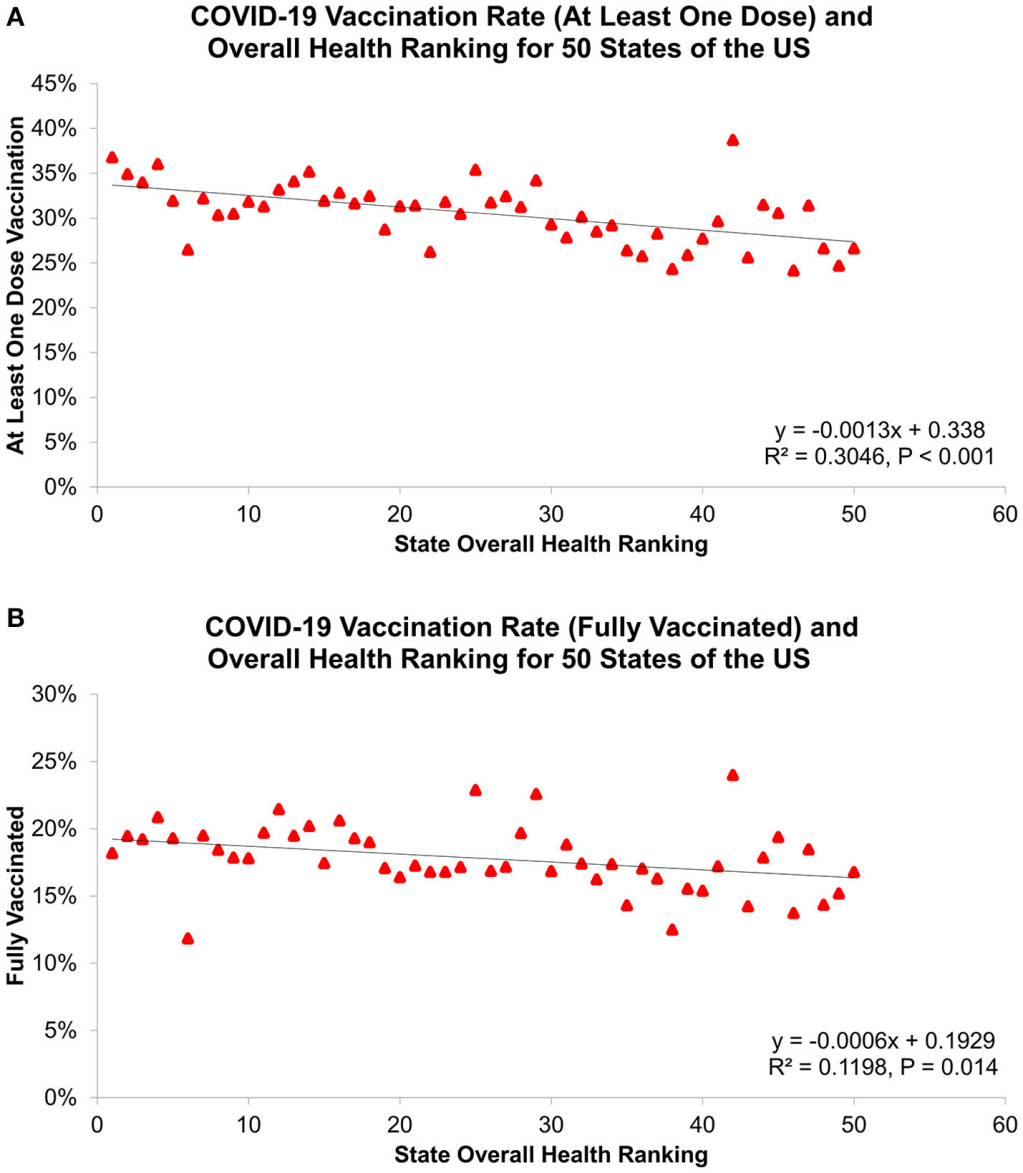
Association of state overall health ranking with COVID-19 vaccination rate for **(A)** as least one dose vaccination and **(B)** fully vaccinated, as of April 1, 2021.

COVID-19 vaccines, with the declaration of Emergency Use Authorization (EUA) by the US Food and Drug Administration (FDA) in December of 2020, have proven to reduce the risk of COVID-19 infection and its potentially severe complications and deaths (39–43). In contrast to the currently available evidence, our study showed no association between COVID-19 vaccination coverage and disease outcomes (the 3rd wave CFR). This result could be explained by the different distribution of time lag between reporting of infections and deaths among states, which affects the estimation of COVID-19 CFRs in the US. According to the CDC, ensuring equitable COVID-19 vaccine access is a priority for the US COVID-19 vaccination program. A recent study by Hughes et al. from CDC COVID-19 Response Team indicated that COVID-19 vaccination coverage was lower in high vulnerability counties than in low vulnerability counties (44). We thus performed a linear regression analysis to examine the relationship between COVID-19 vaccination rates and state health rankings, which showed states with better Overall health rankings were significant associated with higher COVID-19 vaccination coverage (at least one dose: β = −0.13%, R^2^ = 0.305, *P* < 0.001; fully vaccinated: β = −0.06%, R^2^ = 0.120, *P* = 0.014; Figure 8). This finding suggested that COVID-19 vaccination coverage was largely driven by socioeconomic and health disparities and tailored vaccine administration and outreach efforts should be developed to reduce COVID-19 vaccination inequities among states.

The current study is subject to some limitations. First, the CFR analyzed here was based on the reported COVID-19 cases and deaths by states, which is likely to be influenced by the time lags in report dates resulting in a challenge with accurate calculation of the CFR. Second, this cross-sectional, state-level study evaluates population-level risk, but no causal, individual-level inferences can be made, including age, comorbidities, socioeconomic and medical risks. Third, using health factor rankings from AHR to represent the health disparities among states may not be specific. We focused our analysis at the state-level using health indicators of Behaviors, Social and Economic Factors, Physical Environment, Clinical Care, and Health Outcomes, but important variation in socioeconomic and health disparities exists within states and some crucial parameters may not be included. Therefore, the results of this study should be interpreted cautiously, and future research is suggested to strengthen our findings.

In conclusion, this study provided the first state-level assessment and demonstrated substantial geographic differences in COVID-19 CFR in the US. State health rankings, including a wide range of health indicators, were significantly associated with COVID-19 outcomes and vaccination coverage among states. These findings suggested that targeted public health interventions (e.g., bans on public gatherings, stay-at-home policies, and telemedicine), mitigation strategies (e.g., mask use and social distancing) and policies (e.g., COVID-19 vaccination program) addressing health disparities are essential to improve inequitable outcomes of COVID-19 in the US.

## Data Availability Statement

The original contributions presented in the study are included in the article/supplementary material, further inquiries can be directed to the corresponding author/s.

## Author Contributions

Y-CL had full access to all the data in the study and takes responsibility for the integrity of the data and the accuracy of the data analysis. Y-CL, K-YC, and MM contributed to the literature review, study concept and design, data analysis and interpretation, and writing and revision of the manuscript. All authors contributed to the article and approved the submitted version.

## Conflict of Interest

The authors declare that the research was conducted in the absence of any commercial or financial relationships that could be construed as a potential conflict of interest.

## Publisher's Note

All claims expressed in this article are solely those of the authors and do not necessarily represent those of their affiliated organizations, or those of the publisher, the editors and the reviewers. Any product that may be evaluated in this article, or claim that may be made by its manufacturer, is not guaranteed or endorsed by the publisher.

## Abbreviations

AHR: America's Health Rankings
CDC: Centers for Disease Control and Prevention
CFR: Case-Fatality Rate
COVID-19: Coronavirus Disease 2019.
